# Integrating Climate Change Resilience Features into the Incremental Refinement of an Existing Marine Park

**DOI:** 10.1371/journal.pone.0161094

**Published:** 2016-08-16

**Authors:** Harriet N. Davies, Lynnath E. Beckley, Halina T. Kobryn, Amanda T. Lombard, Ben Radford, Andrew Heyward

**Affiliations:** 1 School of Veterinary and Life Sciences, Murdoch University, Perth, Western Australia, Australia; 2 Institute for Coastal and Marine Research, Nelson Mandela Metropolitan University, Port Elizabeth, South Africa; 3 Australian Institute of Marine Science, Perth, Western Australia, Australia; Auckland University of Technology, NEW ZEALAND

## Abstract

Marine protected area (MPA) designs are likely to require iterative refinement as new knowledge is gained. In particular, there is an increasing need to consider the effects of climate change, especially the ability of ecosystems to resist and/or recover from climate-related disturbances, within the MPA planning process. However, there has been limited research addressing the incorporation of climate change resilience into MPA design. This study used Marxan conservation planning software with fine-scale shallow water (<20 m) bathymetry and habitat maps, models of major benthic communities for deeper water, and comprehensive human use information from Ningaloo Marine Park in Western Australia to identify climate change resilience features to integrate into the incremental refinement of the marine park. The study assessed the representation of benthic habitats within the current marine park zones, identified priority areas of high resilience for inclusion within no-take zones and examined if any iterative refinements to the current no-take zones are necessary. Of the 65 habitat classes, 16 did not meet representation targets within the current no-take zones, most of which were in deeper offshore waters. These deeper areas also demonstrated the highest resilience values and, as such, Marxan outputs suggested minor increases to the current no-take zones in the deeper offshore areas. This work demonstrates that inclusion of fine-scale climate change resilience features within the design process for MPAs is feasible, and can be applied to future marine spatial planning practices globally.

## Introduction

Marine protected areas (MPAs), particularly no-take areas, are increasingly considered to be an effective tool to ensure the persistence of healthy marine ecosystems and to increase the resilience of ecological communities [1]. Systematic, quantitative methods [2–4] coupled with robust design criteria which encompass the principles of being comprehensive, adequate, representative, efficient and resilient can facilitate planning of no-take reserves within MPAs to maximise biodiversity conservation outcomes. However, many existing MPAs have been created in an *ad hoc* or opportunistic manner, resulting in some poorly performing or non-representative protected areas [5,6]. In order to achieve conservation goals, some existing MPAs may require incremental refinement as new ecological or socio-economic information becomes available [7].

Coral reefs are considered to be particularly vulnerable to increased disturbance associated with anthropogenic climate change [8]. Specifically, rising sea temperatures can lead to coral bleaching events, and ocean acidification can disturb the chemical processes essential for reef building [8–11]. Whilst these are both processes for which no-take marine reserves cannot mitigate [9,12], it has been found, in some cases, that the presence of well-enforced, no-take marine reserves may reduce the impacts of these threats, thereby increasing the resilience of marine ecosystems to disturbance caused by climate change [10,13]. More importantly, by including the most resilient areas within no-take marine reserves, particularly areas most likely to resist and/or recover from bleaching events, defined biodiversity conservation outcomes are more likely to be achieved into the future [14].

There is currently limited empirical scientific evidence regarding the features determining coral reef resilience to anthropogenic climate change [15]. There are, however, a vast suite of often contradictory hypotheses about which features may contribute to the resilience of coral reefs (Table 1).

**Table 1:** Synthesis of current literature identifying key features which may determine coral reef resilience [15–19].

Resilience Indicator
**Temperature variability** Corals in areas of high thermal variability, or which have shown quick recovery from a thermal stress event, are more likely to be resilient to future events [10,20–24].Areas least exposed to rising temperatures can also be more resilient [25]
**Nutrient loads** High nutrient load from land-based activities, such as agriculture, can cause macro-algal blooms thereby reducing coral resilience [15,26]
**Sedimentation** High levels of sedimentation result in loss of corals and act as a barrier to settlement of coral larvae [27,28]
**Substrate availability** Successful recruitment following disturbance requires suitable hard substrate upon which larvae can settle [29]
**Water mixing** Mixing through waves, currents and upwelling moderates temperatures and reduces extent of coral exposure to thermal stress events [30–32]
**Depth** Coral reefs in deeper water are more likely to resist and recover from disturbance [19]
**Light reduction/ shading** Factors that cool/ shade from high light levels such as reef aspect, mangroves or cliffs can reduce stress on coral [20,33,34]
**Structural complexity** Reefs which exhibit high rugosity and are more structurally complex recover faster than less complex habitats [19]
**Resistant coral forms** Massive corals tend to be more resistant than branching corals and some species appear to be more resilient than others [20,31,35,36]
**Coral diversity** Increased species diversity gives a higher chance of some species surviving and/or recovering from disturbance [15]
**Live coral cover** Live corals that have survived previous stress events are likely to be more tolerant to disturbance and higher densities improve the ability to recover [21,37–39]
**Connectivity** Coral larvae need to be supplied from upstream reefs following severe disturbance [11,40]
**Coral disease** Coral disease can quickly wipe out colonies making recovery less likely [41]
**Macro-algal cover** Areas with high abundance of macro-algae can prevent coral settlement, dominate benthic space and directly kill corals [42,43]
**Herbivore biomass** Herbivores reduce macro-algal cover and ensure bare substrate is available for settlement by coral larvae [29,39,44]
**Fishing pressure** Reduced fishing pressure lowers biological stress on the ecosystem [45]
**Proximity to human activities** Anchor damage, reef walking, boat strikes etc. damage coral and increase susceptibility to disease and bleaching. Proximity to nodes of human activity leads to increased pollution/run off [17,46,47]

It is broadly recognised that conservation planning for MPAs should attempt to incorporate resilience features while remaining adaptive to new research findings [7,48]. In particular, Game et al. [14] argue that in order to achieve the best conservation outcomes, MPAs should be placed in areas most likely to be resilient to disturbance induced by climate change. While the growing need to address climate change resilience within marine conservation planning is well documented [1,7], there are many MPAs that were implemented before it became an important consideration. As such, there have been very few spatially explicit attempts to apply resilience theory to the incremental refinement of existing MPAs [7,11,16–18,49].

**Fig 1:**
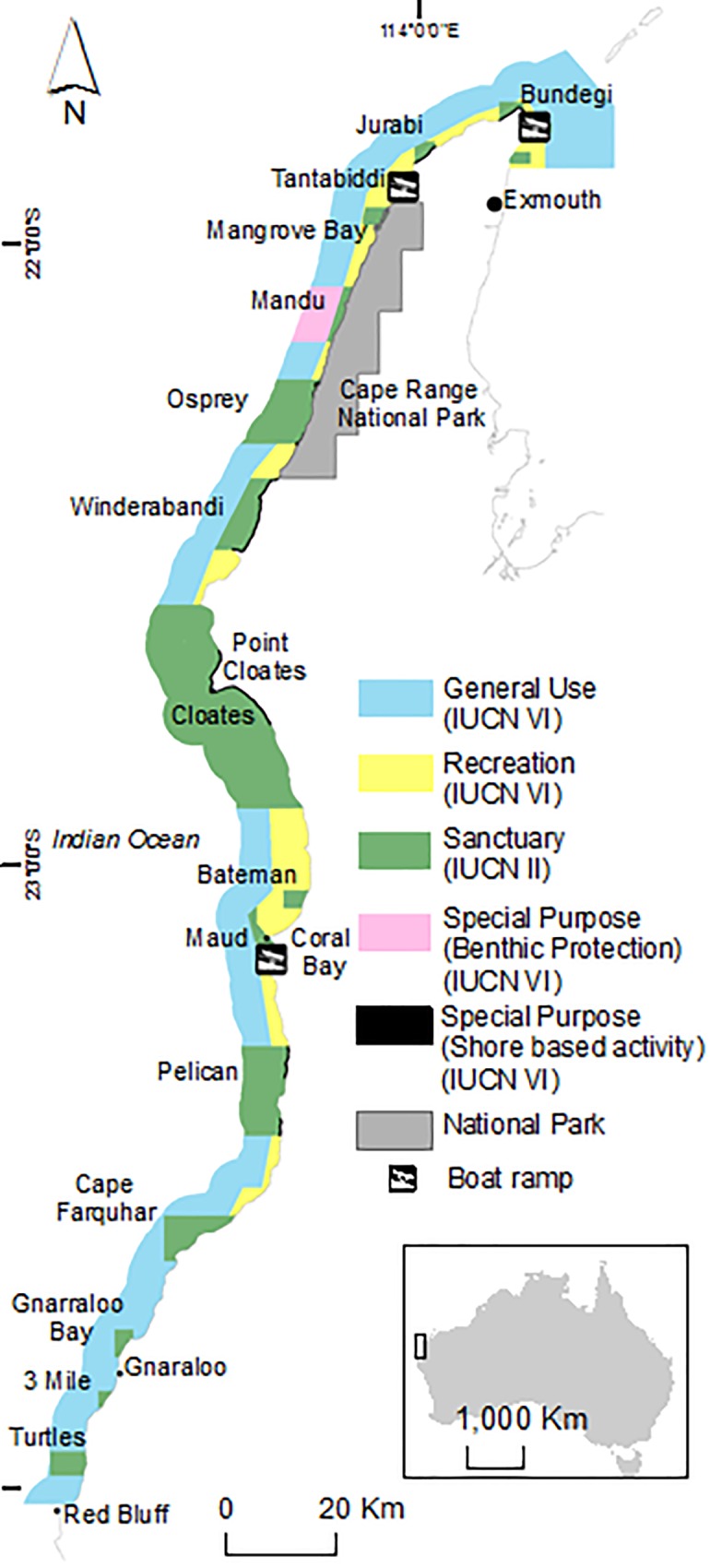
Ningaloo Marine Park indicating management zones: General Use Zone (recreational fishing and limited commercial fishing permitted, IUCN VI), Recreation Zone (recreational fishing permitted, IUCN VI), Special Purpose Zones (recreational shore-based or trolling fishing only, IUCN VI), No-take Sanctuary zones (no fishing permitted, IUCN II).

Ningaloo Marine Park (Fig 1), in Western Australia, is a well-established MPA and UNESCO World Heritage Site with relatively few ecosystem threats in comparison with other coral reefs [50,51]. Following a recent severe ocean warming event which resulted in significant coral bleaching [36,52–54], rising sea surface temperatures (SSTs) associated with climate change are expected to present the greatest threat to this ecosystem. However, the existing management plan does not explicitly consider climate change.

Recent investment in research within the Park has resulted in spatially explicit datasets, which have provided an excellent opportunity to classify climate change resilience features, and to demonstrate one approach for integrating these features into an incremental refinement of the Park’s zonation within the existing adaptive management framework. The aims of this study were to identify any gaps in representation of benthic habitats within existing no-take sanctuary zones; to define and map climate change resilience features and to assess their representation in existing no-take sanctuary zones; and to conduct a spatial analysis using Marxan software to make incremental refinements of existing zones to ensure that the benthic and climate change resilience features are adequately represented within no-take sanctuary zones.

## Materials & Methods

### Study region

Ningaloo Reef (Fig 1) on Australia’s north-west coast is one of the largest fringing coral reefs in the world [50]. At ~300 km long, it spans three degrees of latitude from 21°S to 24°S, encompasses a diverse range of habitats and has high species richness [55,56]. The reef crest forms a discontinuous barrier along the coast, creating a lagoon with an average width of 2.5 km [57]. At the southern and northern extents of the reef, the lagoons disappear and are replaced by extensive intertidal reef platforms [57]. The reef crest provides a buffer to the prevailing south-westerly winds and waves and is irregularly interspersed by reef passes allowing water circulation in and out of the lagoons [57,58]. The lagoons display high annual sea surface temperature variability of over 10°C in comparison to the relatively more stable temperatures within reef pass systems [59].

In 2011, Ningaloo Reef experienced an extreme marine heat wave event driven by some of the strongest La Niña conditions in the past century [53,60]. High sea level anomalies, increased cyclonic activity and record high water temperatures resulted in mass coral mortality [36,52], with up to 95% loss of coral cover and the complete loss of *Acropora* and *Montipora* assemblages in some areas [36]. There has been a discernible increase in the occurrence of SST anomalies, with higher than average ocean temperatures observed in the two austral summers following the 2011 event [54,61], and the most extreme historical anomalies recorded post-1980 [61].

Ningaloo Marine Park is a popular tourism destination with approximately 240,000 visitors annually [62]. There is extensive recreational fishing and some commercial charter fishing activity, but no other commercial fisheries currently operate within the marine park [63].

### Datasets

Datasets were acquired from both published and unpublished sources, were all spatially explicit and, when combined, covered the entire extent of the Ningaloo Marine Park (Table 2).

**Table 2:** Details of datasets used for the incremental refinement of Ningaloo Marine Park no-take sanctuary zones to accommodate resilience features.

Data type		Description	Source
**Biodiversity**For classes see [S1 Table]	Shallow water habitats (<20 m)	HyMap airborne hyperspectral imagery at 3.5 × 3.5 m resolution. Habitats separated into 46 biotic and abiotic classes	[64]
	Deeper water benthic communities	Deeper water habitat models with 19 biotic classes at 100 × 100 m resolution	[65]
**Physical**	Bathymetry (depth, rugosity)	HyMap airborne hyperspectral imagery (125 bands) at 3.5 × 3.5 m resolution	[64]
	Geomorphic features	Digitized polygons of reef passes, lagoon areas and reef crests	This study
	Coastline	Line file depicting mean high water line of Ningaloo coast (1:100,000)	[66]
**Human use/ Cost**	Boat-based activities	Distribution and density of boat- based activities over year in 3 × 3 km grid	[67]
	Shore-based activities	Distribution and density of shore-based recreational activities over year in 3 × 3 km grid	[67]
	Camp sites and access points	Distribution and density of camp sites and boat launch sites over year in 3 km coastal segments	[68]
	Commercial fisheries	Catch and effort of charter fishing from 2009–2013 in 10 × 10 nautical mile data blocks	WA Department of Fisheries (unpublished data)
**Other**	Current Ningaloo Marine Park management zones	Polygon file depicting spatial boundaries of each zone type	[69]

The study area was divided into 1 km^2^ planning units in a grid format [4] using ArcGIS 10.2 software [70]. This layer was overlaid with each of the defined datasets (Table 2) to determine areas of the features present within each planning unit.

### Benthic biodiversity

The shallow water marine habitats of Ningaloo to 20 m depth have been mapped using HyMap airborne hyperspectral imagery at 3.5 m pixel resolution [64]. For the deeper water benthic communities, spatial habitat models were constructed using towed video camera imagery of the benthos in depths from 15–130 m throughout the marine park captured together with single beam echo-sounder transects [65] (Table 2) (For detailed methods see S2).

### Resilience features

Six resilience features were selected from the available data (Table 2) on the basis of their relevance to Ningaloo Reef and the importance inferred by relevant literature (Table 3, for rationale see S3 Table).

**Table 3:** Six climate change resilience features highly relevant for Ningaloo Marine Park, and the definition of resilience values assigned to each feature (Percentage values for macro-algal and live coral cover obtained from Kobryn et al. [64]).

		Resilience value		
Resilience feature	Priority for Ningaloo Reef	High (1)	Moderate (2)	Low (3)
**Depth**	Areas deeper than 8 m [19,39]	>8 m	6–8m	<6 m
**Structural complexity**	Structurally complex areas with high rugosity values [19]	Rugosity value 4–5	Rugosity value 2–3	Rugosity value 0–1
**Water mixing**	Reef pass areas with high mixing [15]	Reef pass present	N/A	No reef pass present
**Macro-algal cover**	Areas with low macro-algal cover [13]	Sparse macro-algal cover (<35%)	Patchy macro-algal cover (35–65%)	Dominant macro-algal cover (>65%)
**Live coral cover**	Areas with high live coral cover [16,39]	Continuous coral(>65%)	Patchy coral(35–65%)	Sparse coral(<35%)
**Proximity to human activities**	Areas furthest from human activity nodes [10,39]	Low human activity	Moderate human activity	High human activity

The 3.5 m pixel HyMap bathymetry data [64] layer was utilised to map the water depth throughout the extent of the study area. It was also utilised to create a rugosity map, as a surrogate for habitat complexity, using the Benthic Terrain Modeller Tool for ArcGIS which creates an output of rugosity from digital elevation models. The rugosity values were reclassified into six structural complexity classes following similar criteria to those used by Graham et al. [19] namely, 0 = no vertical relief, 1 = low and sparse relief, 2 = low but widespread relief, 3 = widespread moderately complex relief, 4 = widespread very complex relief and 5 = exceptionally complex relief.

Reef passes, as an indicator for water mixing, were also determined through use of the 3.5 m pixel HyMap bathymetry data [64] and manually digitized in ArcGIS. Reef passes were defined as an opening between reef crests where depths >5 m extended landward beyond the reef crest into the lagoon. Reef passes are <1 km wide; any gaps >1 km in width were considered to be larger reef breaks and are less likely to experience the strong current flow characteristic of reef passes at Ningaloo Reef [58]. Percentage cover of macro-algae and live coral was determined using the 3.5 m resolution shallow water benthic habitat maps which were limited to <20 m depth [64]. Proximity to human activities [67] was calculated using the cost layer outlined below, and the normalised values were subsequently split into equal terciles of high, moderate and low.

Each feature had to meet specific conditions to be assigned a resilience value of 1 for high resilience, 2 for moderate resilience and 3 for low resilience (Table 3). The planning unit grid file was overlaid with each resilience feature layer to create six GIS layers with a resilience value for each 1 km^2^ planning unit. Any planning units that fell outside the extent of the resilience feature datasets were assigned a score of 1 (high resilience) as they all occurred in deeper water (>20 m) [19].

A test for correlation between each feature found high structural complexity to be strongly correlated with low macro-algal cover (R^2^ = 0.98) and high live coral cover (R^2^ = 0.77). High live coral cover and low macro-algal cover also had a positive correlation (R^2^ = 0.67). These strong correlations could enable the resilience index to be simplified with high structural complexity acting as a surrogate with minimal reductions in accuracy. However, retaining the correlated features increased the irreplaceability of the planning units which contained all three features. With the ultimate aim of incorporating the planning units with the highest resilience within the reserve network, retaining all six features was considered preferable.

### Cost data

Boat-based and shore-based human activity data collected through aerial surveys and presented in 3 × 3 km data blocks [67,68] were utilised to derive a spatially explicit socio-economic cost layer. Only peak visitor period data were used and both data files were clipped to the extent of the Ningaloo Marine Park boundary with the shore-based data aggregated to be included within the coastal planning units.

In addition, catch and effort data of commercial charter fishing activities from 2009 to 2014 in 10 × 10 nautical mile data blocks were provided by the Western Australian Department of Fisheries. Total catch values were summed for all years and clipped to the extent of the planning unit layer.

Data detailing distribution of individual recreational activities such as fishing, diving and relaxing enabled a comprehensive socio-economic cost layer to be developed [67]. Values from each of the cost datasets (Table 2) were normalised and fishing values were then multiplied by 10 because displacement of fishing by no-take zones has the highest opportunity cost [71]. All other values were left on a scale of 0–1 as the activities would not be displaced by the presence of a no-take sanctuary zone (S4 Table). Each of the cost data layers was then overlaid with the planning unit grid layer and summed to assign a single opportunity cost to each 1 km^2^ planning unit.

### Incorporating resilience features into conservation planning

A commonly used systematic conservation planning software, Marxan (version 2.43) [72], was used to incorporate the resilience values into a conservation planning exercise. Marxan is an open access software designed to solve the minimum set reserve design problem [73]. It uses a simulated annealing algorithm with spatially derived planning units to find a set of near optimal reserve solutions which minimise socio-economic cost while meeting user-defined biodiversity targets (i.e. conservation features) [74].

A boundary length file was created using a tool developed by ABPmer Marine Environmental Research [75] in ArcGIS and a boundary length modifier of 0.001 was determined using the methods of Stewart and Possingham [76].

### Conservation objectives

Current international guidelines recommend that a target of 30–40% minimum representation of conservation features be included within no-take sanctuary zones [1,77]. The existing management plan for Ningaloo Marine Park did not define any quantitative targets for the inclusion of specific habitat types within no-take sanctuary zones [69] and the final boundaries of these zones were delineated by negotiation with stakeholders, resulting in 34% of the total area of the Park being designated as no-take sanctuary zones. However, these zones are biased towards certain habitats and are unrepresentative of others [4]. In order to quantify this problem and recommend redress, we formulated a desired target of 34% representation within sanctuary zones of each benthic biodiversity feature. The target was based on the international guidelines previously mentioned, as well as the stakeholder negotiation previously undertaken in the Park.

With the same rationale used to define 34% targets for habitat types, a 34% target for features conferring high resilience was also used, assuming that these features represent areas most likely to resist and/or recover from future thermal disturbances and are therefore critical areas that require adequate representation within no-take sanctuary zones. Following examination of the literature, six relevant studies were chosen to identify the relative importance of each resilience feature ([15–19,39] (see S3 Table for rationale). As the perceived importance of individual features varied between authors, we used the same (34%) target for each resilience feature.

### Analysis

ArcGIS was used to calculate the percentage of shallow water habitats and deeper water benthic communities currently represented within no-take sanctuary zones in the Marine Park. Marxan analyses, following standard methods [78], were then undertaken to identify additional priority areas for inclusion within no-take sanctuary zones that would enable the 34% representation targets to be met. For each of the analyses, 10,000 repetitions and two million iterations were used and a high penalty value was set for each feature to ensure all objectives were achieved. The six resilience features were incorporated as additional conservation features similar to the method used by Levy and Ban [12]. All analyses included the same socio-economic cost layer.

To explore the influence of existing sanctuary zones, distribution of biodiversity features and areas of high resilience on Marxan outputs, six scenarios with different combinations of variables were analysed. These were; S1. Biodiversity features only; S2. Biodiversity features with existing sanctuary zones; S3. Biodiversity and resilience features; S4. Biodiversity and resilience features with existing sanctuary zones; S5. Resilience features only and S6. Resilience features with existing sanctuary zones.

A complete hierarchical cluster analysis based on a Jaccard resemblance matrix similar to the method of Harris et al. [79] was performed in R version 3.2.5 to compare within, and among, the scenarios using the top 100 solutions. Cohen’s Kappa statistic was also used to make pairwise comparisons of the ‘best’ Marxan solutions from each scenario.

Marxan generates a summed solution output for each scenario which provides the selection frequency of each planning unit across all 10,000 repetitions. With a focus on iterative refinement of the existing no-take zones, these outputs for scenarios S2 and S4, and the difference between the two scenarios, were plotted in ArcGIS to determine which planning units had the highest selection frequency and, therefore, were most likely to be present in a final representative reserve design. In order to compare different design options, Marxan calculates an objective function score, which combines the boundary length, any penalties for not meeting targets, and the total cost for the reserve network [74]. The ‘best’ solution output is the run with the lowest objective function value and can be considered a near optimal solution within a suite of other options [78]. The ‘best’ solution outputs were plotted in ArcGIS and total area of the ‘best’ recommended reserve system calculated.

### Limitations

Small gaps existed in the benthic datasets, which meant those areas would have been less likely to be included in the reserve network by Marxan. A number of studies have also incorporated SST variables when determining areas of thermal refugia for inclusion within reserves [10,25]. Although SST data could be useful to delineate thermal refuges in deeper environments, the resolution of remotely sensed SST data is relatively coarse and can be inaccurate in the shallow, nearshore and narrow lagoons found at Ningaloo. As the benthic datasets used in this study are very high resolution, in this instance, including SST data would detract from the high level of spatial resolution provided by the benthic datasets and thus was not included in this analysis.

## Results

### Habitat representation in no-take zones

Of the 46 shallow water habitat classes identified in the Ningaloo Marine Park [64], 45 had >40% representation within current no-take sanctuary zones. The only habitat missing the target (patchy tabulate coral) accounted for a very small proportion of total habitat coverage (<100 m^2^) and it is only found within the southern regions of the reef. Of the 16 deeper water benthic community classes, three were represented at >34%, while most classes required small increases (1–6%) in representation within sanctuary zones. However, to attain the 34% target, a >10% increase in area within no-take sanctuary zones is required for dense, sparse and medium filter feeder communities and dense sponge communities.

### Resilience

Planning units considered to be highly resilient comprised 1360 km^2^ of the total Marine Park and, of those, 15% fell within existing no-take zones. There was a trend towards higher resilience values being located in offshore areas with moderate resilience values generally associated with the reef crest region and lowest resilience in the inshore, shallower areas.

**Fig 2:**
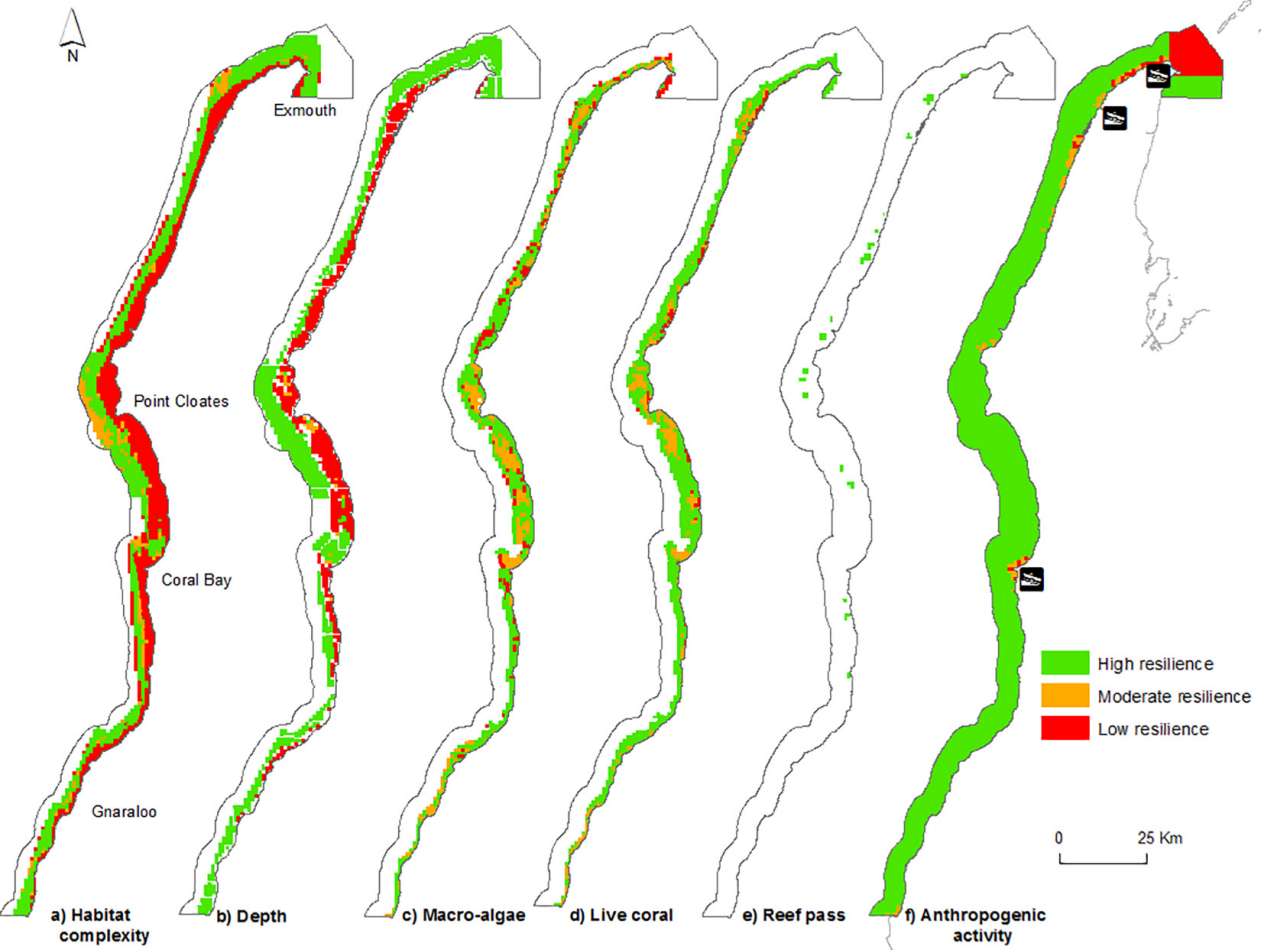
Spatial distribution of resilience features at Ningaloo Marine Park; a) Structural complexity, b) Depth, c) Live coral cover, d) Macro-algal cover, e) Reef passes (water mixing), f) Proximity to anthropogenic activity.

The greatest structural complexity was generally found seaward of the reef crest (Fig 2A). Depth increased with distance from shore although there were some small, deeper areas inside the reef crest (Fig 2B). Areas with high macro-algal cover (low resilience value) were restricted to small areas close to the coast (Fig 2C), whilst continuous live coral cover was evenly distributed throughout the marine park (Fig 2D). Reef passes were distributed throughout most of the marine park, although they diminish in the southern areas where the lagoon systems disappear (Fig 2E). High human impacts were generally located near the two major hubs of Coral Bay and Exmouth with moderate levels throughout the coastal area of Cape Range National Park (Fig 2F).

### Summary of Marxan solutions

When ignoring the existing zones, the target of 34% representation of biodiversity features (S1) could be achieved with 28.3% of the NMP demarcated as sanctuary zones (Table 4).

**Table 4:** Summary of conditions set and Marxan outputs for 6 different scenarios tested where a target of 34% representation for each feature within each scenario was met. Note that the total reserve area of existing sanctuary zones is 884 km^2^ (34%) and total area of Ningaloo Marine Park is 2633 km^2^.

Scenario	Variables			Marxan ‘best’solution	
	Biodiversity features	Resilience features	Existing sanctuaries locked in?	Total sanctuary zone area (% of NMP)	Change in no-take zone area (% change)
**S1 Biodiversity only**	Yes	No	No	746.6 km^2^ (28.3%)	-137.4 km^2^(-5.2%)
**S2 Biodiversity & zones**	Yes	No	Yes	1204.6 km^2^ (45.8%)	+320.6 km^2^ (+12.1%)
**S3 Biodiversity & resilience**	Yes	Yes	No	1262.2 km^2^ (47.9%)	+378.2 km^2^ (+14.3%)
**S4 Biodiversity, resilience & zones**	Yes	Yes	Yes	1404.2 km^2^ (53%)	+518.2 km^2^(+19.6%)
**S5 Resilience only**	No	Yes	No	1085.8 km^2^ (41.2%)	+201.8 km^2^ (+7.6%)
**S6 Resilience & zones**	No	Yes	Yes	1087.1 km^2^ (41.0%)	+197.1 km^2^ (+7.4%)

An iterative refinement of the existing zones (S2- Biodiversity & zones) required an increase in the area of no-take sanctuary zones by 12.1% to meet the same objectives. Adding the resilience targets required an increase in no-take sanctuary zones of 14.3% without locking in the existing zones (S3) and an iterative refinement with resilience features (S4) required a total of 53% of the NMP to be demarcated as no-take sanctuary zones (Table 4).

**Fig 3:**
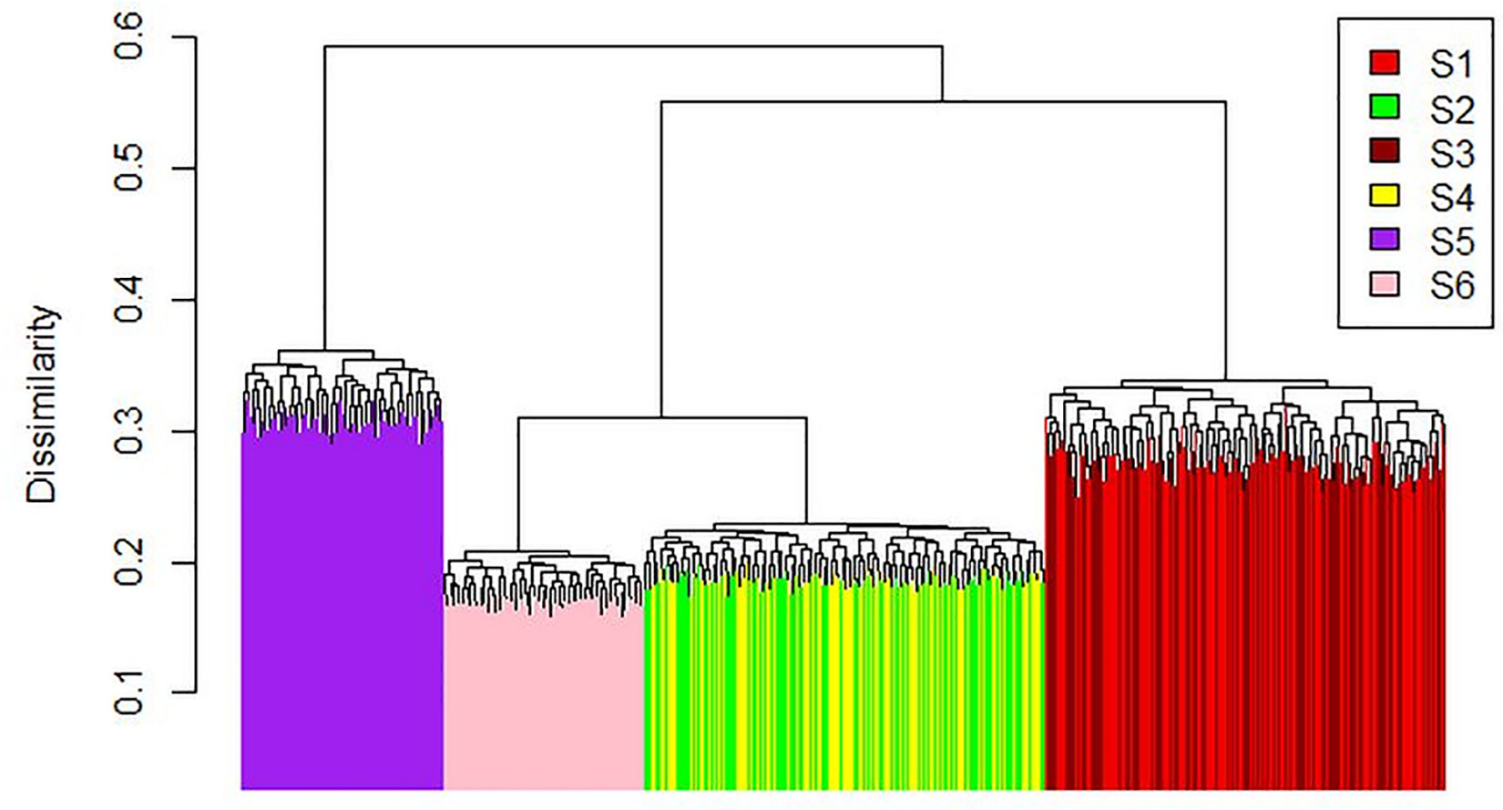
Dendrogram from a complete hierarchical cluster analysis based on a Jaccard resemblance matrix. S1–S6 refer to the six scenarios: S1 Biodiversity features only, S2 Biodiversity features with existing sanctuary zones, S3 Biodiversity and resilience features, S4 Biodiversity and resilience features with existing sanctuary zones, S5 Resilience features only and S6 Resilience features with existing sanctuary zones.

The complete hierarchal cluster analysis of the top 100 solutions per scenario showed S5 (resilience features only without existing zones) was the most dissimilar to the other scenarios (Fig 3). The second split was driven by whether the scenarios had existing sanctuary zones locked in. Finally, within the three scenarios that did have existing sanctuary zones locked in, S6 (resilience only) was separated from the scenarios which included the biodiversity targets (Fig 3). The solutions generated by S1 and S3 and by S2 and S4, respectively showed no dissimilarity thus, for NMP, adding the resilience targets had little bearing on the Marxan solutions.

With a focus on adaptive management of the existing Marine Park through the incremental refinement of the existing zones, results from scenarios S2 and S4 were examined in more detail.

### Incremental refinement of the existing Marine Park

**Fig 4:**
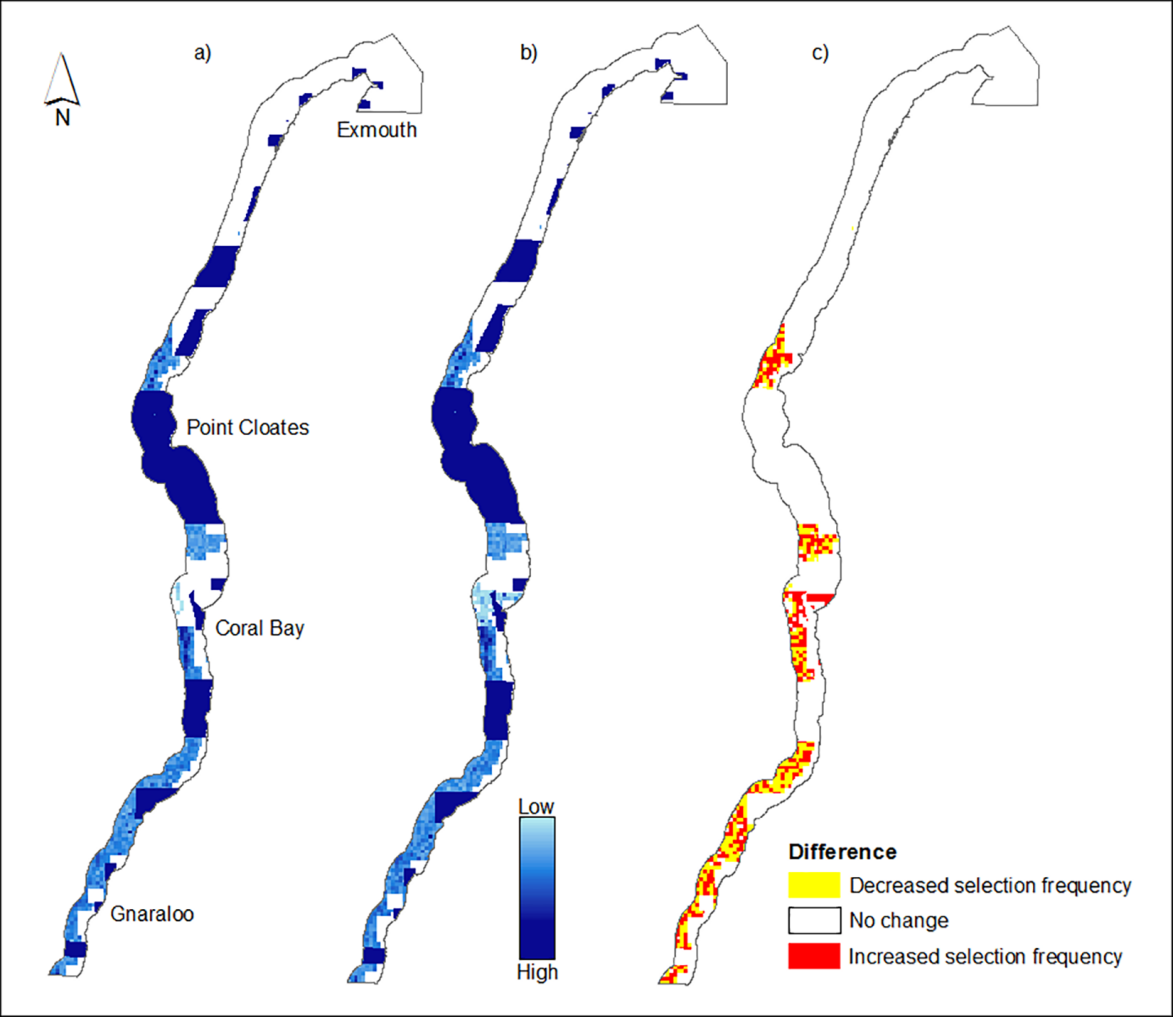
a) Selection frequency of Ningaloo Marine Park planning units to achieve 34% target representation for all biological conservation features in an incremental refinement of existing no-take sanctuary zones (Scenario S2), b) Selection frequency of Ningaloo Marine Park planning units to achieve 34% target representation for all biological conservation features and resilience features in an incremental refinement of existing no-take sanctuary zones (Scenario S4), c) Difference in selection frequency of Ningaloo Marine Park planning units between scenario S2 and scenario S4 (S2 subtracted from S4).

The summed solution output for the S2 scenario identified areas of high selection frequency offshore in the region north of Point Cloates and in the southern reaches offshore from Gnaraloo (Fig 4A). There were a few planning units of high selection frequency in the northern region of the Marine Park corresponding to the area adjacent to Cape Range National Park and Exmouth and relatively evenly distributed areas of high selection frequency outside sanctuary zones throughout the rest of the Marine Park (Fig 4A). When the high resilience features were added as conservation features (Scenario S4), the summed solution output (Fig 4B) was only marginally different to the S2 scenario (Cohens Kappa = 0.798). The greatest increase in selection frequency following the addition of resilience features was off the coast of Coral Bay (Fig 4C).

**Fig 5:**
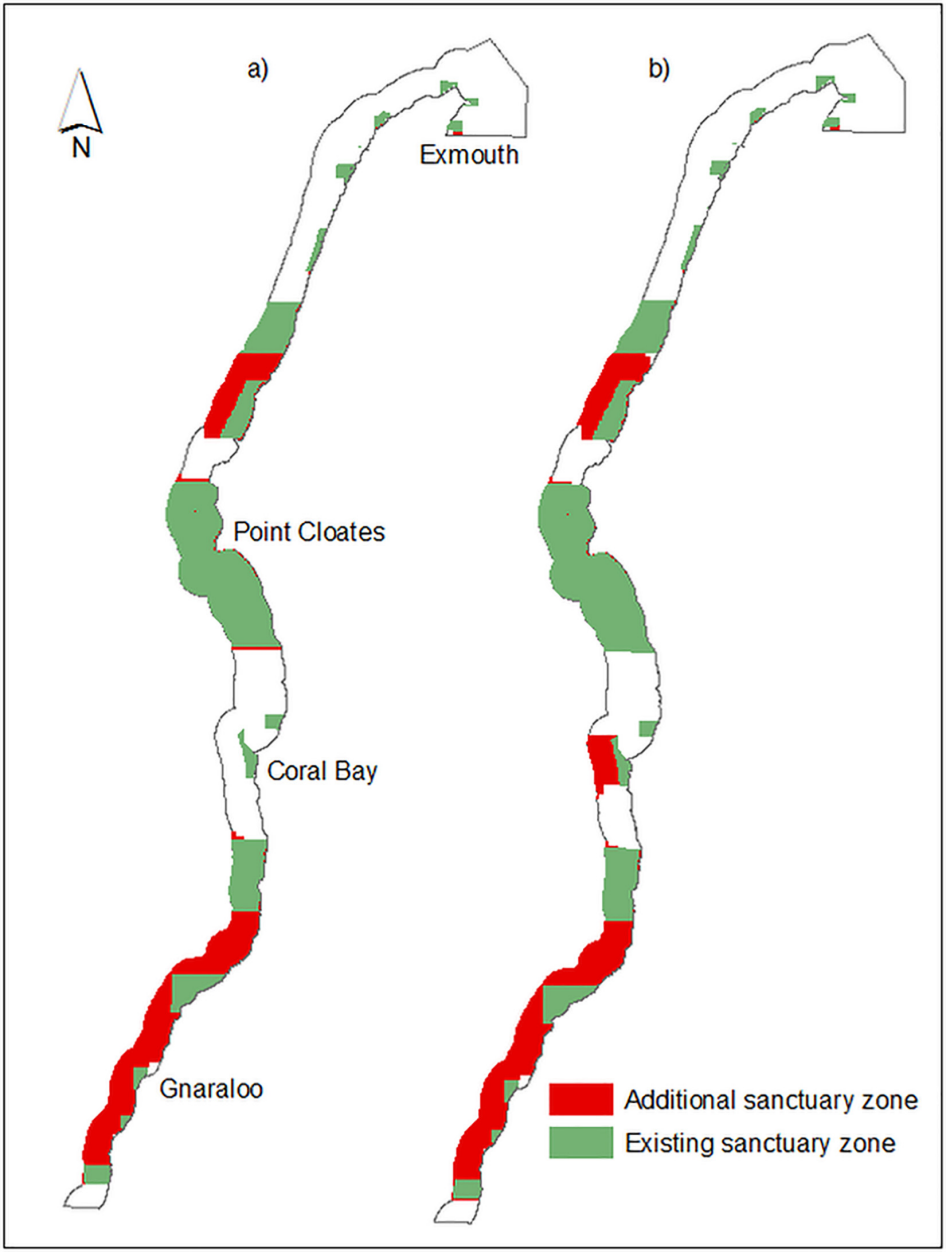
a) MARXAN ‘best’ reserve design output meeting 34% target representation for all biological conservation features in an incremental refinement of existing no-take sanctuary zones (Scenario S2), b) MARXAN ‘best’ reserve design output meeting 34% target representation for all biological conservation features and resilience features, in an incremental refinement of existing sanctuary zones (Scenario S4).

In scenario S2, the southern regions were represented within the ‘best’ reserve design output as two large sanctuary zones with all additional zones increasing the current no-take sanctuary area by 320.2 km^2^ (12.1%) (Fig 5A). When the resilience features were added (scenario S4), the ‘best’ reserve design was very similar to the output of S2, however, there was one major addition to the sanctuary zone network offshore from Coral Bay which resulted in a further increase in sanctuary zone area of 197.6 km^2^ (Fig 5B). The Marxan ‘best’ outputs for both scenarios also added the existing shore-based activity special purpose zones (narrow shore-side strips) to the no-take sanctuary network (Fig 5A & 5B).

## Discussion

### Method for incorporating climate change resilience features

As many existing marine parks have been established for several decades, there is a growing need for methods which enable an assessment of their management zones against the most recent scientific information, particularly with respect to climate change. This study demonstrates a method by which climate change resilience features can be defined, delineated and then incorporated into the incremental refinement of an existing marine reserve network for coral reefs and contributes to the limited research on the topic [7,80]. The availability of remotely-sensed, hyperspectral data provided a good opportunity to isolate resilience features at a high resolution over a large area. It provided resilience information on a much finer scale than similar research using remote sensing products [12,17], yet without the limited spatial cover associated with fine scale in-water research [18,19]. Further analyses could incorporate fine-scale resilience features with the spatial distribution of past thermal stress [10], models of predicted SSTs into the future [81] or both [12,25]. The method could also be developed to provide insights into marine spatial planning with multiple zones [25], and could consider the level of connectivity between more resilient and less resilient areas [82,83].

This method for developing a spatial distribution of resilience levels from remote sensing products can inform management decisions beyond MPAs through highlighting vulnerable areas that may require other management measures [84]. The depth limitations of hyperspectral imagery (<20 m) were overcome by combining the shallow water dataset with the acoustic single-beam dataset from deeper water. While the single beam dataset has a lower resolution, it enables a comprehensive analysis for the entire extent of the marine park.

### Resilience of Ningaloo Reef

Unusually, Ningaloo Reef exhibits a naturally high level of macro-algal cover within the lagoon where both coral and macro-algal habitats exist side by side [85]. This is typically an indicator of a reef in decline, however, high macro-algal cover is considered to be a healthy stable state for Ningaloo Reef [86]. Despite the naturally high occurrence, a recent study indicated that high macro-algal cover still impedes coral larvae settlement at Ningaloo [87] and therefore reduces the ability for corals to recover following a bleaching event. Consequently, naturally high macro-algal cover, coupled with shallower water and less water mixing within the lagoon systems is a likely driver of the lower coral resilience values found in the lagoon areas.

### Refinement of Ningaloo Marine Park

The Ningaloo Marine Park management plan is now due for a ten-year review. Investment in research since the establishment of the Marine Park in its current form has resulted in extensive, spatially-explicit, ecological and socio-economic data enabling a robust adaptive management planning process.

The current no-take sanctuary zones have some shortfalls in representation, particularly in deeper, offshore areas. However, all conservation features can be met through incremental increases to existing sanctuary zones in two main regions, namely the seaward extension of the Winderabandi sanctuary zone out to the Ningaloo Marine Park boundary and extending and connecting the very small sanctuary zones from Cape Farquhar to 3 Mile sanctuary in the south, confirming the recommendations of Beckley and Lombard [4].

The suggested increase in sanctuary representation in offshore areas is aligned with global trends where offshore and pelagic waters are often underrepresented within MPAs [88,89]. The biodiversity benefits of increased offshore sanctuary zones could be further enhanced through connectivity with the proposed offshore Commonwealth sanctuary zones within the Exclusive Economic Zone [90]. Furthermore, with the highest densities of human activity occurring within inshore areas, incremental increases to sanctuary zones in offshore regions will enable conservation features to be met with low socio-economic cost [91].

The large reserve area recommended in the south could further be beneficial to the resilience of the Ningaloo Marine Park because of the predicted range shift to higher latitudes of marine species in various climate change scenarios for Western Australia [92]. Furthermore, the regions of the reef at higher latitudes, while still expected to experience a gradual increase in temperature over time, are less likely to experience severe pulse type thermal stress events [60]. With recent research indicating that some coral families can acclimatise to gradually rising temperatures [24], these southern areas may be even more important for persistence than the resilience index implies.

Some highly resilient areas were represented within the current sanctuary zones. However, the shallow water habitat data were collected before the 2011 and subsequent 2012/13 bleaching events and, as such, the benthic composition and coral cover may have changed. For example, within the Bundegi sanctuary zone (Fig 1) 90% of live coral cover was lost [36]. As such, in order to ensure effective adaptive management, a repeat survey, using the same hyperspectral and acoustic methods, could be implemented and included within the resilience framework developed by this study.

Incremental reserve design has been found to be an effective tool for adaptive management [93]. However, this study found similar results to Stewart et al. [94], whereby incremental refinement required a larger area to meet targets than if a systematic conservation planning process was used without considering the existing zoning. While Airamé et al. [95] suggested removal of historic reserves if they were not included within systematic solutions, longevity is important for the success of reserves [96], and increases in the biomass of certain fish species within the current reserves at Ningaloo have already been recorded [97]. Therefore, an iterative refinement of the existing no-take sanctuary zones is the best option for a more representative reserve system that encompasses areas critical for resilience to climate change induced disturbance.

Incorporating resilience features as additional conservation objectives gives managers some assurance that a reserve network might have the best possible chance of achieving biodiversity outcomes in the face of climate change [14,49]. In some cases, achieving this objective might result in a very different reserve system, however, for Ningaloo Marine Park, ensuring that the most resilient areas of the reef were represented within the reserve network could be achieved with only minor additions to the representative no-take sanctuary zones.

### Resilience and marine reserves

There is some empirical evidence to suggest that coral reefs within marine reserves may be more resilient to the impacts of climate change [98]. Some areas within reserves have demonstrated an enhanced ability to recover following extreme weather events [99] and Caribbean coral reefs within reserves were found to have significantly better recovery rates after a bleaching event than reefs without reserve protection [13]. The ability for reserves to increase ecosystem resilience to climate change beyond the reserve boundaries themselves is likely to be linked to how well the populations within reserves can enhance the recovery of downstream degraded areas through connectivity [83].

Following the extreme marine heat wave event at Ningaloo Reef in 2011, bleaching was indiscriminate when it came to reserves [36,52], and it will take many years to determine if the areas within the reserves display enhanced recovery. Furthermore, the presence or absence of marine reserves was found to have no bearing on the resistance or recovery of coral reefs in the Seychelles following an extreme bleaching event in 1998 [19]. However, poaching and illegal fishing in these reserves is common, in which case, the benefits from reserve protection would not be apparent [100,101].

Although the role no-take marine reserves play in increasing the resilience of reefs is still uncertain, and likely to be linked to a number of other variables [102], there are many measurable benefits directly linked to no-take reserves [103,104]. The prioritisation of the representation of highly resilient areas, or known areas of refuge to thermal stress, within reserve networks is therefore a sound precautionary principle and fundamental to achieve biodiversity conservation outcomes [81,105].

### Conclusions

Consideration of the predicted impacts of climate change in conservation planning is critical, yet, practical application is scarce. This study demonstrates that isolating features likely to confer resilience on a coral reef is feasible and, with the use of hyperspectral remote-sensing and modern acoustics, it can be achieved at high resolution across large study areas. As more empirical evidence regarding the factors that make coral reefs resilient becomes available, conservation planners can further refine resilience features and incorporate areas of high conservation significance within planned MPAs in a relatively straightforward manner. While it is still difficult to predict the impacts of climate change with any degree of certainty, it is imperative that resilience is incorporated into MPAs in order to have the highest probability of long-term persistence.

## Supporting Information

S1 Table Habitat classes of shallow and deep water benthic biodiversity datasets. (DOCX)

S2 Table A) Datasets derived from single beam bathymetry that were used as environmental variables for modelling biota, substrate and fish abundance/richness, B) Model accuracy statistic AUC statistic for biotic and abiotic substrate predicted from the presence/absence models (blind validation n = 19872 data points).
(DOCX)

S3 Table Rationale for developing resilience conservation objectives. (DOCX)

S4 Table Opportunity cost values for different human activities within Ningaloo Marine Park. (DOCX)
