# Supplementary material for: Integrating Climate Change Resilience Features into the Incremental Refinement of an Existing Marine Park
Source: PLoS One. 2016 Aug 16;11(8):e0161094. doi: 10.1371/journal.pone.0161094 (PMC4986976; doi:10.1371/journal.pone.0161094)
Supplement: S1 Table — (DOCX) [file pone.0161094.s001.docx]

# S1. Shallow water < 20 m habitat classes defined by Kobryn et al. (2013)

| F | Habitat classes |
| --- | --- |
| 1 | Dominant limestone pavement with sand |
| 2 | Sparse macro-algae with sand |
| 3 | Dominant hard coral with turf or macro-algae-covered dead coral or rubble (hard coral = Continuous tabulate coral) |
| 4 | Dominant hard coral with limestone pavement (hard coral = Dominant tabulate coral with digitate coral, encrusting coral and soft coral) |
| 5 | Dominant hard coral with sand (hard coral = Continuous blue -tip coral) |
| 6 | Sparse hard coral with limestone pavement (hard coral = Patchy digitate coral and tabulate coral) |
| 7 | Sparse hard coral and macro-algae with limestone pavement and sand (hard coral = Continuous digitate coral) |
| 8 | Sparse hard coral, macro-algae and turf algae with limestone pavement (hard coral = Continuous digitate coral) |
| 9 | Sparse macro-algae with limestone pavement |
| 10 | Sparse macro-algae with limestone pavement and sand |
| 11 | Sparse turf algae with limestone pavement |
| 12 | Dominant macro-algae with limestone pavement |
| 13 | Dominant macro-algae with rubble |
| 14 | Dominant macro-algae with sand |
| 15 | Patchy hard coral with turf or macro-algae-covered dead coral or rubble (hard coral = Continuous digitate coral) |
| 16 | Patchy hard coral with sand (hard coral = Continuous tabulate coral) |
| 17 | Patchy limestone pavement, rubble and sand |
| 18 | Patchy hard coral with turf or macro-algae-covered dead coral or rubble (hard coral = Continuous tabulate coral) |
| 19 | Patchy limestone pavement with sand |
| 20 | Patchy hard coral with turf or macro-algae-covered dead coral or rubble and sand (hard coral = Dominant Blue Tip coral with sparse digitate coral) |
| 21 | Patchy hard coral with turf or macro-algae-covered dead coral or rubble and sand (hard coral = Continuous Blue Tip coral) |
| 22 | Patchy hard coral with limestone pavement (hard coral = Dominant tabulate coral with sparse digitate coral) |
| 23 | Patchy hard coral with limestone pavement (hard coral = Continuous digitate coral) |
| 24 | Patchy hard coral with limestone pavement (hard coral = Continuous tabulate coral) |
| 25 | Patchy hard coral with sand (hard coral = Patchy branching coral, tabulate coral, foliaceous coral, massive coral and sub-massive coral) |
| 26 | Patchy hard coral with turf or macro-algae-covered dead coral or rubble (hard coral = Patchy digitate coral and tabulate coral) |
| 27 | Patchy hard coral with turf or macro-algae-covered dead coral or rubble (hard coral = Continuous Blue Tip coral) |
| 28 | Patchy hard coral with turf or macro-algae-covered dead coral or rubble (hard coral = Continuous massive coral) |
| 29 | Continuous sand |
| 30 | Patchy macro-algae with limestone pavement |
| 31 | Patchy macro-algae with limestone pavement and sand |
| 32 | Patchy macro-algae with sand |
| 33 | Continuous limestone pavement light |
| 34 | Continuous limestone pavement dark |
| 35 | Continuous soft coral |
| 36 | Dominant soft coral, sparse hard coral and macro-algae with limestone pavement (hard coral = Continuous digitate coral) |
| 37 | Continuous hard coral (hard coral = Continuous blue tip coral) |
| 38 | Continuous hard coral (hard coral = Continuous foliaceous coral) |
| 39 | Dominant soft coral, sparse hard coral with limestone pavement (hard coral = Continuous digitate coral) |
| 40 | Continuous hard coral (hard coral = Dominant tabulate coral with sparse digitate coral) |
| 41 | Continuous hard coral (hard coral = Dominant tabulate coral with sparse massive coral and submassive coral) |
| 42 | Continuous hard coral (hard coral = Continuous branching coral) |
| 43 | Continuous hard coral (hard coral = Continuous digitate coral) |
| 44 | Continuous hard coral (hard coral = Continuous tabulate coral) |
| 45 | Continuous hard coral (hard coral = Continuous massive coral) |
| 46 | Continuous macro-algae |
| 47 | Unclassified |

# Deeper water benthic community habitat types classified by Colquhoun et al. (2007)

|  | Benthic community type |
| --- | --- |
| 1 | Alcyon |
| 2 | Anemone |
| 3 | Ascidian |
| 4 | Bryozoan |
| 5 | Burrowers |
| 6 | Crinoids |
| 7 | Sea Fans |
| 8 | Filterers |
| 9 | Dense Filterers |
| 10 | Medium Filterers |
| 11 | Sparse Filterers |
| 12 | Gorgonians |
| 13 | Dense Hard Coral |
| 14 | Medium Hard Coral |
| 15 | Hydroids |
| 16 | Medium Macro-algae |
| 17 | Rhodoliths |
| 18 | Dense Sponge |
| 19 | Medium Sponge |
