# Supplementary material for: Integrating Climate Change Resilience Features into the Incremental Refinement of an Existing Marine Park
Source: PLoS One. 2016 Aug 16;11(8):e0161094. doi: 10.1371/journal.pone.0161094 (PMC4986976; doi:10.1371/journal.pone.0161094)
Supplement: S2 Table — A) Datasets derived from single beam bathymetry that were used as environmental variables for modelling biota, substrate and fish abundance/richness, B) Model accuracy statistic AUC statistic for biotic and abiotic substrate predicted from the presence/absence models (blind validation n = 19872 data points). (DOCX) [file pone.0161094.s002.docx]

S2 Supplementary Methods

Deep water benthic communities

The single beam acoustic data were interpolated to generate a digital elevation model (DEM) covering the length of Ningaloo Reef at a 100 m^2^ grid pixel resolution. Spatial habitat models were constructed using the towed video data as the dependent variable with the DEM plus derivatives as predictor variables.  These models were constructed using logistic regression following similar methods to Holmes et al. [1]. However, there were two major differences, specifically, comparison of grid pixels where habitat was present versus pixels where habitat was not found (i.e. not based on video points of presence and absence) and secondly, logistic regression was used rather than regression trees.

S2A Table. Datasets derived from single beam bathymetry that were used as environmental variables for modelling biota, substrate and fish abundance/richness.

| **Predictor datasets** | **Definition** |
| --- | --- |
| Bathymetry | All elevation is relative to the Australian Height Datum (AHD) tidally corrected to mean sea level (MSL) |
| Aspect | Azimuthal direction of the steepest slope, calculated on a 3 x 3 m pixel area |
| Slope | First derivative of elevation: Average change in elevation / distance calculated on a 3 x 3 m pixel area |
| Profile curvature | Second derivative of elevation: concavity/convexity parallel to the slope, calculated on a 3 x 3 m pixel area |
| Plan curvature | Second derivative of elevation: concavity/convexity perpendicular to the slope, calculated on a 3 x 3 m pixel area |
| Curvature | Combined index of profile and plan curvature |
| Local relief (Range) ^a^ | Maximum minus the minimum elevation in a local neighbourhood |

^a^ Local neighbourhood analysis: run on circles of kernel pixel radius 3 original cell size is 50m

1. Holmes KW, Van Niel KP, Radford B, Kendrick GA, Grove SL. Modelling distribution of marine benthos from hydro-acoustics and underwater video. Cont Shelf Res. 2008; 28: 1800–1810.

S2B Table Model accuracy statistic AUC statistic for biotic and abiotic substrate predicted from the presence/absence models (blind validation n = 19872 data points). D = dense cover >40%, M = medium cover 11-39% and S= sparse cover 1-10%.

| **Group** | **AUC** | **Testing N** | **Probability cut-off** |
| --- | --- | --- | --- |
| Bryozoan | 0.9644 | 91 | 0.1616 |
| Crinoids | 0.9878 | 43 | 0.0757 |
| Crustacean | 0.9121 | 45 | 0.2987 |
| Sea Fans S | 0.8188 | 21 | 0.913 |
| Macro-algae/Filter Feeder D | 0.9653 | 78 | 0.9624 |
| Macro-algae/Filter Feeder M | 0.8876 | 90 | 0.1463 |
| Macro-algae/Filter Feeder S | 0.9018 | 156 | 0.1935 |
| Macro-algae M | 0.941 | 13 | 0.4241 |
| Macro-algae S | 0.9382 | 56 | 0.3775 |
| Soft Coral D | 0.913 | 28 | 0.1985 |
| Soft Coral M | 0.927 | 35 | 0.3525 |
| Soft Coral S | 0.9213 | 81 | 0.3888 |
| Sponge D | 0.834 | 112 | 0.4615 |
| Sponge M | 0.853 | 108 | 0.3966 |
| Sponge S | 0.8677 | 238 | 0.8736 |
| Sea whips D | 0.9282 | 472 | 0.6095 |
| Sea whips M | 0.8723 | 779 | 0.4053 |
| Sea whips S | 0.8266 | 439 | 0.3004 |
| Gorgonians M | 0.8891 | 12 | 0.4597 |
| Gorgonians S | 0.9448 | 6 | 0.4935 |
| Halimeda | 0.6516 | 15 | 0.323 |
| Hard Coral D | 0.6812 | 144 | 0.2512 |
| Hard Coral & Filter Feeders D | 0.9481 | 57 | 0.7721 |
| Hard Coral & Filter Feeders M | 0.9506 | 165 | 0.7402 |
| Hard Coral & Filter Feeders S | 0.9204 | 140 | 0.1024 |
| Hard Coral M | 0.9535 | 39 | 0.3143 |
| Hard Coral S | 0.9567 | 287 | 0.9566 |
| Hard Coral & Sponge M | 0.9965 | 10 | 0.3226 |
| Hydroids S | 0.9542 | 18 | 0.8402 |
| Isolates S | 0.7403 | 101 | 0.6518 |
| Limestone reef (cover 11-39%) | 0.9536 | 68 | 0.7676 |
| Limestone reef (cover >= 40%) | 0.9695 | 107 | 0.8131 |
| Limestone reef (cover 1-9%) | 0.9495 | 72 | 0.9428 |
