# Supplementary material for: Integrating Climate Change Resilience Features into the Incremental Refinement of an Existing Marine Park
Source: PLoS One. 2016 Aug 16;11(8):e0161094. doi: 10.1371/journal.pone.0161094 (PMC4986976; doi:10.1371/journal.pone.0161094)
Supplement: S3 Table — (DOCX) [file pone.0161094.s003.docx]

| S3 Table Developing conservation objectives for level of representation of resilience features within no-take sanctuary zones. | | | | | | | | | |
| --- | --- | --- | --- | --- | --- | --- | --- | --- | --- |
| **Resilience Indicator** | **Justification** | **Conditions for high resilience** | **Authors perceived importance**  **C=critical, VI=very important, I=important** | | | | | | **Conservation objective for Ningaloo Reef- Representation within no-take sanctuary zones** |
|  |  |  | [1] | [2] | [3] | [4] | [5] | [6] |  |
| Depth | Empirical evidence from Seychelles and Scott Reef off Western Australia demonstrates that corals in deeper water are more resistant to thermal stress and more likely to recover [5,6] | Deeper coral reefs |  |  |  |  | C | C | ≥34% deeper (>8m) areas |
| Habitat complexity | Empirical evidence following a bleaching event in Seychelles suggests high habitat complexity is critical for resistance and recovery [5] | High habitat complexity |  | VI |  | VI | C |  | ≥34% areas with high rugosity |
| Macro-algal cover | Areas with high levels of macro-algae can prevent coral settlement, dominate benthic space and directly kill corals [7,8] Ningaloo has naturally high macro-algal cover so these areas will be limited. | Low macro-algal cover | C | C |  |  |  |  | ≥34% areas with low macro-algal cover |
| Proximity to anthropogenic activities | Anchor damage, people standing on reef, boat strikes damage coral and increase susceptibility to disease and bleaching, proximity to hubs of human activity results in increased pollution etc. [9–11] | Further from human impacts | VI | C | C | VI |  | C | ≥34% areas furthest from high levels of human activity |
| Water mixing | Mixing keeps temperatures relatively constant and reduces extent of shallow water coral exposure to thermal stress [12–15] | More mixing | VI | VI |  | C |  |  | ≥34% of reef passes |
| Live coral cover | Indicative of past stress tolerance, many reefs are self-recruiting so if some die the survivors can recolonise [6,16–18] | High coral cover | C | VI | I | I | VI | C | ≥34% areas with high live coral cover |
