# Supplementary material for: Integrating Climate Change Resilience Features into the Incremental Refinement of an Existing Marine Park
Source: PLoS One. 2016 Aug 16;11(8):e0161094. doi: 10.1371/journal.pone.0161094 (PMC4986976; doi:10.1371/journal.pone.0161094)
Supplement: S4 Table — (DOCX) [file pone.0161094.s004.docx]

S4 Table. Scaling factors for opportunity cost values used for different human activities within Ningaloo Marine Park.

| Activity | Cost |
| --- | --- |
| Recreational fishing | x10 |
| Commercial charter fishing | x10 |
| Wildlife interaction | x1 |
| Scuba diving | x1 |
| Snorkelling | x1 |
| Surfing | x1 |
| Relaxing on the beach | x1 |
